# AGREEing on clinical practice guidelines for idiopathic steroid-sensitive nephrotic syndrome in children

**DOI:** 10.1186/s13643-021-01666-w

**Published:** 2021-05-10

**Authors:** Khalid Abdulaziz Alhasan, Reem Al Khalifah, Majed Aloufi, Weiam Almaiman, Muddathir Hamad, Naif Abdulmajeed, Abdullah Al Salloum, Jameela A. Kari, Muneera AlJelaify, Rolan K. Bassrawi, Turki Al Hussain, Adi Alherbish, Abdulhadi Al Talhi, Mohamad-Hani Temsah, Sidharth Kumar Sethi, Rupesh Raina, Reny Joseph, Yasser Sami Amer, on behalf of the Saudi Society of Nephrology and Transplantation

**Affiliations:** 1 Pediatric Nephrology Unit, Pediatrics Department, College of Medicine, King Saud University, Riyadh, Saudi Arabia; 2 Saudi Society of Nephrology and Transplantation (SSN&T), Riyadh, Saudi Arabia; 3 Pediatric Endocrinology Unit, Pediatrics Department, College of Medicine, King Saud University, Riyadh, Saudi Arabia; 4 Department of Pediatric Nephrology, Prince Sultan Military Medical City, Riyadh, Saudi Arabia; 5 College of Medicine, Alfaisal University, Riyadh, Saudi Arabia; 6 Nephrology Section, Pediatrics Department, King Faisal Specialist Hospital and Research Center, Riyadh, Saudi Arabia; 7 Pediatric Neurology Unit, Pediatrics Department, King Khalid University Hospital, King Saud University Medical City, Riyadh, Saudi Arabia; 8 Prince Sultan Military Medical City, Riyadh, Saudi Arabia; 9 Pediatric Nephrology Division, Department of Pediatrics, Pediatric Nephrology Center of Excellence Faculty of Medicine, King Abdulaziz University, Jeddah, Saudi Arabia; 10 Pharmacy Services Department, King Khalid University Hospital, King Saud University Medical City, Riyadh, Saudi Arabia; 11 General Pediatrics Unit, Pediatrics Department, King Khalid University Hospital, King Saud University Medical City, Riyadh, Saudi Arabia; 12 Department of Pathology and Laboratory Medicine, King Faisal Specialist Hospital and Research Center, Riyadh, Saudi Arabia; 13 Pediatrics Department, College of Medicine, King Saud University, Riyadh, Saudi Arabia; 14 Department of Pediatric Nephrology, Pediatrics Hospital, King Saud Medical City, Riyadh, Saudi Arabia; 15 Imam Mohammed Bin Saud University, Riyadh, Saudi Arabia; 16 Pediatric Intensive Care Unit, Pediatric Department, College of Medicine, King Saud University, Riyadh, Saudi Arabia; 17 Pediatric Nephrology, Medanta, The Medicity, Gurgaon, India; 18 Pediatric Nephrology, Akron Children’s Hospital, Akron, OH USA; 19 Ward 11B and Day Care, Pediatrics-Nursing, King Khalid University Hospital, King Saud University Medical City, Riyadh, Saudi Arabia; 20 Pediatrics Department, King Khalid University Hospital, Riyadh, Saudi Arabia; 21 Clinical Practice Guidelines Unit, Quality Management Department, King Saud University Medical City, Riyadh, Saudi Arabia; 22 Research Chair for Evidence-Based Health Care and Knowledge Translation, King Saud University, Riyadh, Saudi Arabia; 23 Alexandria Center for Evidence-Based Clinical Practice Guidelines, Alexandria University, Alexandria, Egypt

**Keywords:** Nephrotic syndrome, Pediatrics, Clinical practice guidelines, Systematic review, AGREE II Instrument, Quality assessment

## Abstract

**Background:**

Nephrotic syndrome is the most common kidney disease in children worldwide. Our aim was to critically appraise the quality of recent Clinical Practice Guidelines (CPGs) for idiopathic steroid-sensitive nephrotic syndrome (SSNS) in children in addition to summarize and compare their recommendations.

**Methods:**

Systematic review of CPGs. We identified clinical questions and eligibility criteria and searched and screened for CPGs using bibliographic and CPG databases. Each included CPG was assessed by four independent appraisers using the Appraisal of Guidelines for REsearch & Evaluation II (AGREE-II) instrument. We summarized the recommendations in a comparison practical table.

**Results:**

Our search retrieved 282 citations, of which three CPGs were eligible and appraised: Kidney Disease: Improving Global Outcomes (KDIGO) 2012, Japan Society for Pediatric Nephrology (JSPN) 2014, and American Academy of Pediatrics (AAP) 2009. Among these, the overall assessment of two evidence-based CPGs scored > 70% (KDIGO and JSPN), which was consistent with their higher scores in the six domains of the AGREE II Instrument. In domain 3 (rigor of development), KDIGO, JSPN, and AAP scored 84%, 74%, and 41%, respectively. In domain 5 (applicability), they scored 22%, 16%, and 19%, respectively, and in domain 6 (editorial independence), they scored 94%, 65%, and 88%, respectively.

**Conclusions:**

The methodological quality of the KDIGO CPG was superior, followed by JSPN and AAP CPGs with the relevant recommendations for use in practice.

**Systematic review registration:**

The protocol was registered in the Center for Open Science (OSF) DOI: 10.17605/OSF.IO/6QTMD and in the International prospective register of systematic reviews PROSPERO 2020 CRD42020197511.

**Supplementary Information:**

The online version contains supplementary material available at 10.1186/s13643-021-01666-w.

## Background

Nephrotic syndrome is considered the most common kidney disease in children worldwide. It is defined by a clinical characteristic of hypoalbuminemia < 25 g/L, edema and nephrotic range proteinuria > 40 mg/m^2^/h, or protein/creatinine ratio > 200 mg/mmol in a spot urine sample [1, 2]. There are many classifications of nephrotic syndrome: one of the classifications is based on the clinical response to steroids. Most children with nephrotic syndrome respond to steroids within 4 weeks of proper steroid therapy (i.e., steroid-sensitive nephrotic syndrome [SSNS]); however, these children behave differently afterward [3].

The Kidney Disease: Improving Global Outcomes (KDIGO) stated the important scientific definitions of nephrotic syndrome: Patients with steroid-dependent nephrotic syndrome (SDNS) are defined as patients who have relapse while weaning the steroid dose or within 14 days of steroid discontinuation. Frequently relapsing nephrotic syndrome (FRNS) is defined as two or more relapses in 6 months after the initial response or four and more relapses in any 1-year period [2].

The global incidence rate of nephrotic syndrome of childhood is variable among countries and ranges from 1.15 to 16.9 per 100,000 children annually [4, 5]. Children with nephrotic syndrome require prolonged use of immunosuppressive agents, with multiple adverse effects, including infections and other side effects. A study conducted in a tertiary care center in Saudi Arabia by Alfakeeh et al. showed that the cumulative yearly dose of steroids is an important independent risk of infection [6].

In our practice, we noted center-to-center differences in managing patients diagnosed with SSNS, SDNS, and FRNS. The main differences we observed were in the duration of steroid therapy, steroid weaning, selection of second-line immunosuppressive agent and its targeted levels, and other practice parameters [7–9].

We would like to adapt a unified national evidence-based clinical practice guideline (CPG) for the management of these patients. Our aim from developing this CPG is to unify the practice between centers and improve patients’ outcomes and experience.

CPGs are statements that include recommendations intended to optimize patient care that are informed by a systematic review (SR) of evidence and an assessment of the benefits and harms of alternative care options [10]. To date, there are no national CPGs to provide evidence-based guidance for healthcare professionals during the provision of clinical care for children with idiopathic SSNS in Saudi Arabia. In 2019, a decision was made to launch a project for adaptation of a national evidence-based CPG for the management of children with SSNS by the Department of Pediatrics, College of Medicine, King Saud University (KSU) in collaboration with the Saudi Society of Nephrology and Transplantation, as the governing body of nephrology including pediatric nephrology practice in Saudi Arabia, to provide guidance and recommendations to pediatricians, nephrologists, pharmacists, nurses, pathologists, children with SSNS, and all related stakeholders in Saudi Arabia who care for these children. The project is guided by the “KSU-Modified-ADAPTE” as a formal CPG adaptation methodology consisting of three phases: setup, adaptation, and finalization [11–13].

The Appraisal of Guidelines for REsearch & Evaluation (AGREE II) instrument is the gold standard for the quality appraisal of CPGs. AGREE II is a validated CPG appraisal tool cited in > 1013 articles and endorsed by several healthcare organizations [14–16]. AGREE II identifies components that should be addressed by CPGs to improve their quality and trustworthiness and obtain positive patient outcomes [11, 14–16].

Since the SR of CPGs, using AGREE II, is a key step in the CPG adaptation process, we have dedicated this study to report the results of this SR and critically appraise recently published CPGs for childhood SSNS using AGREE II [11, 17, 18].

We utilized the PIPOH Model [i.e., Population (P), Intervention (I), Professionals (P), Outcomes (O), and Healthcare setting or context (H)] [11, 12, 17] where the *Population* (*P*) included children aged 2–12 years with non-congenital, idiopathic SSNS, including new-onset nephrotic syndrome, SDNS, or FRNS without any comorbidities. Intervention(s) (I) included all pharmacological therapeutic agents. *Professionals* (*P*) or target users of CPGs included mainly pediatric nephrologists, general pediatricians, and pharmacists and nurses with relevant nephrology experience. *Outcomes* (*O*) included prevention of disease relapse, appropriateness of prescription (i.e., duration of steroid courses in newly diagnosed SSNS and drug of choice of the second agent in SDNS or FRNS). *Healthcare settings or context* (*H*) included CPGs to be used in secondary and tertiary healthcare settings. The four main health questions were prioritized for this review. Additionally, we have utilized the PICAR statement where P: Population, clinical indication(s), and condition(s), I: Intervention(s), C: Comparator(s), Comparison(s), and (key) Content, A: Attributes of eligible CPGs, and R: Recommendation characteristics [17].

### Health questions

1. Among children aged 2–12 years with noncongenital, idiopathic SSNS, what is the preferred and best effective treatment to prevent disease relapse?
2. Among children aged 2–12 years with SSNS, what is the appropriate steroid and duration of the steroid course in newly diagnosed children with SSNS?
3. Among children aged 2–12 years with FRNS or SDNS, what is the most appropriate drug as the second-line agent to induce disease remission?
4. Among children aged 2–12 years with non-congenital, idiopathic SSNS, what is the preferred genetic testing to be conducted?

## Methods

The protocol for this study was registered in PROSPERO (International Prospective Register of Systematic Reviews) (Protocol ID: CRD42020197511) and in the Center for Open Science (OSF) (DOI 10.17605/OSF.IO/6QTMD).

Our CPG working group included expert pediatric nephrologists, general pediatricians, a pediatric intensivist, a clinical pharmacist, a renal pathologist, and a specialized nurse guided by two pediatricians with expertise in CPG methodologies. Two external international experts in nephrology were invited as international collaborators to contribute to this CPG project.

### Data sources and search strategy

We systematically searched MEDLINE and EMBASE databases for relevant guidelines using the Ovid platform and hand-searched EBSCO DynaMed Plus (USA), ECRI Guidelines Trust, Guidelines International Network, International Guideline Library, National Institute for Health and Care Excellence (UK), Scottish Intercollegiate Guidelines Network (UK), and Australian National Health and Medical Research Council (Australia). Moreover, we searched databases of national and international societies specializing in fields related to our health topic of SSNS, including the Japanese Society of Pediatric Nephrology (JSPN), KDIGO, International Society of Nephrology, American Society of Pediatric Nephrology, National Kidney Foundation, American Academy of Pediatrics (AAP), and Scottish Paediatric Renal and Urology Network. The search terms used included combinations of subject headings and keywords with various synonyms for idiopathic SSNS, nephrotic syndrome, nephrology, pediatrics, pediatric medicine, child health, treatment, management, pharmacology, practice guidelines, CPGs, healthcare quality, patient safety, evidence-based medicine, AGREE II instrument, quality assessment, critical appraisal, and evidence-based pediatrics (see search strategy in additional file 1). The search was limited to published or updated CPGs between January 1, 2009, and December 31, 2019. We have decided on the last 10 years as the cutoff for dates of publication because typically CPGs are updated every 2–5 years [19]. The search was conducted by two CPG methodologists (RA and YA). We utilized the PIPOH model in addition to the PICAR statement (additional file 2) to support the CPG eligibility identification process [11, 12, 17]. Two reviewers (MA and AA) independently screened titles and abstracts of retrieved CPGs and articles meeting the inclusion criteria. The screening and full-text review were checked by three different reviewers (MH, AA, and AA). Disagreements were resolved by focus group discussions after retrieving and reviewing the full-text articles or full CPG documents.

### Inclusion and exclusion criteria

Teams of two reviewers, independently and in duplicate, screened titles and abstracts and potentially eligible full-text reports to determine eligibility. Disagreements were resolved through a review by RA. The eligibility criteria were as follows: (1) evidence-based with a clear record of their development methods; (2) English or Arabic language; (3) original source CPGs (de novo development); (4) national or international scope; and (5) published by an organization or group authorship and accessible from a CPG database or peer-reviewed journal. Only the most current version of each source CPG was appraised.

The exclusion criteria were CPGs that were published earlier than 2009, not in the English or Arabic language, adapted from other CPGs, presented as consensus or expert-based statements, or had a single author.

### AGREE II instrument workshop

The two CPG methodologists (YA and RA) conducted a capacity building workshop for the review team through hands-on sessions in the concepts of evidence-based medicine and evidence-based CPG standards using the AGREE II instrument tool in 2 days. During the workshop, participants refined the research questions of interest to adapt a CPG to local practice (see the abovementioned health questions). Afterward, each reviewer scored his/her assigned CPGs. All four reviewers critically appraised each CPG. All appraisers reviewed the full CPG documents, including any updates with any relevant supplementary information or links to online web pages related to the CPG methods or CPG implementation tools. For each item, AGREE appraisers were instructed to record the justifications for their scores in the “Comment” section [20].

### Assessment of childhood SSNS CPGs using AGREE II

The AGREE II instrument (www.agreetrust.org) consisted of 23 items organized into six domains: scope and purpose, stakeholder involvement, rigor of development, clarity of presentation, applicability, and editorial independence [14, 15]. Each item was scored on a 7-point Likert scale. The AGREE II evaluation was guided by utilizing its online version: “My AGREE PLUS,” which supports having a CPG appraisal group for each CPG that compiles and calculates the items’ ratings into domain ratings and comments [14, 15]. The four AGREE II appraisers for each CPG comprised a multidisciplinary group with expertise in pediatric nephrology (consultant physicians and head nurse) and pediatric clinical pharmacology (one clinical pharmacist), in addition to a general pediatrician with expertise in CPG methodologies.

Wide discrepancies between the assessors’ scores of items or questions (i.e., whenever there was a difference between these scores of > 3) were resolved by discussion with the appraisal group. The online My AGREE PLUS automatically calculated the standardized AGREE domain scores or ratings (%). We agreed upon a cutoff point of 70% for each AGREE standardized domain score or rating. After the appraisal, more weight was emphasized on the scores of domains 3 and 5 to facilitate the filtration and final evaluation of the reporting quality of included CPGs. Similar cutoff values were reported [21–23]. In addition to the classification of the six AGREE II domains, the evidence base of the included CPGs, their references’ sections, was screened for SRs or meta-analyses, specifically Cochrane reviews.

### Analysis plan

For each AGREE II domain, we calculated standardized scores ranging from 0 to 100% using the methods suggested by the AGREE II instrument. The key recommendations of the eligible CPGs were summarized in a comparative tabular format. The quality of CPGs was classified based upon the rating of domain 3 (rigour of development) where a high-quality CPG will receive a standardized domain rating of more than or equal to 70%, moderate quality CPG (40–69%), and low quality (less than 40%).

## Results

### Identification of CPGs for SSNS in children

**Fig. 1:**
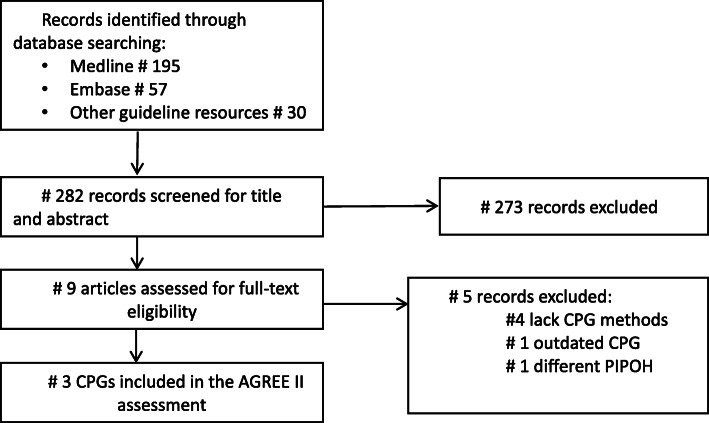
Systematic search and selection of the clinical practice guidelines for management of idiopathic SSNS in children [Moher 2009]. For more information, visit www.prisma-statement.org

We retrieved a total of 282 records. After screening titles and abstracts, eight were included for full-text assessment, and only three were eligible for the review as illustrated in the PRISMA [24] flowchart (Fig. 1) and the PRISMA checklist (additional file 3). These CPGs were developed by the AAP [25], JSPN [26–28], and KDIGO–Chapter 3 [29]. At the time of writing this manuscript, the 2020 KDIGO “CPG on Glomerular Diseases” update was under development as the public review has just closed in the official KDIGO website [30]

### Key characteristics of childhood SSNS CPGs

Table 1 highlights the characteristics of all eligible CPGs. The CPG developer organizations were reference, specialized professional organizations in pediatrics or nephrology, including KDIGO, AAP, and JSPN. All organizations were from high-income countries.

**Table 1:** Characteristics of the included childhood SSNS CPGs

Title	Year of publication	Country (economic level)	Methods of development	Total number of CSRs
AAP—Management of childhood onset nephrotic syndrome	2009	USA (high-income country)	Consensus-based with literature review	1
JSPN—Evidence-based clinical practice guidelines for nephrotic syndrome	2014	Japan (high-income country)	MINDS, GRADE	6
KDIGO—Clinical practice guideline for glomerulonephritis—Chapter 3	2012	International (not applicable)	GRADE	4

*CPG* clinical practice guideline; *CSR* Cochrane systematic review; *GRADE* Grading of Recommendations, Assessment, Development and Evaluations; *MINDS* Medical Information Network Distribution Service

### Reporting the quality of Childhood SSNS CPGs

The AGREE II standardized domain ratings are summarized in Table 2.

**Table 2:** AGREE II standardized domain scores for childhood SSNS CPGs

CPGs/AGREE II domain-standardized scores (%)	AAP 2009 [25]	JSPN 2014 [26–28]	KDIGO 2012 [29]
Domain 1. Scope and Purpose Items 1–3: Objectives; Health question(s); Population (patients, public, etc.)	75%	65%	100%
Domain 2. Stakeholder Involvement Items 4–6: Group Membership; Target population preferences and views; Target users	60%	86%	64%
Domain 3. Rigor of development Items 7–14: Search methods; Evidence selection criteria; Strengths and limitations of the evidence; Formulation of recommendations; Consideration of benefits and harms; Link between recommendations and evidence; External review; Updating procedure.	41%	74%	84%
Domain 4. Clarity and presentation Items 15–17: Specific and unambiguous recommendations; Management options; Identifiable key recommendations	78%	90%	100%
Domain 5. Applicability Items 18–21: Facilitators and barriers to application; Implementation advice/ tools; Resource implications; Monitoring/auditing criteria	19%	16%	22%
Domain 6. Editorial independence Items 22, 23: Funding body; Competing interests	88%	65%	94%
Overall Assessment 1 (Overall quality)	58%	71%	75%
Overall Assessment 2 (Recommend the CPG for use by four appraisers)	Yes (*n* = 0); Yes with modifications (*n* = 3); No (*n* = 1).	Yes (*n* = 1); Yes with modifications (*n* = 3); No (*n* = 0).	Yes (*n* = 2); Yes with modifications (*n* = 2); No (*n* = 0).

*AGREE* Appraisal of Guidelines for REsearch & Evaluation, *AAP* American Academy of Pediatrics, *CPGs* Clinical Practice Guidelines, *JSPN* Japanese Society of Pediatric Nephrology, *KDIGO* Kidney Disease: Improving Global Outcomes, *SSNS* steroid-sensitive nephrotic syndrome

### Domain 1: scope and purpose

The AGREE II standardized score for domain 1 ranged from 65 to 100%. The scores of two CPGs were > 70% in domain 1 (KDIGO = 100% and AAP = 75%).

### Domain 2: stakeholder involvement

The AGREE II standardized domain scores for domain 2 ranged from 60 to 86%. The score of a single CPG was > 70% in domain 2 (JSPN = 86%).

### Domain 3: rigor of development

The AGREE II standardized scores for domain 3 ranged from 41 to 84%. The score of two CPGs were > 70% in domain 3 (KDIGO = 84% and JSPN = 74%). They both reported utilizing the Grading of Recommendations, Assessment, Development and Evaluations method. Moreover, the KDIGO CPG reported its adherence to two sets of CPG standards, namely the Conference on Guideline Standardization Checklist for Reporting CPGs and the Institute of Medicine Standards for Systematic Reviews and Guidelines.

### Domain 4: clarity of presentation

The AGREE II standardized scores for domain 4 ranged from 78 to 100%. The scores of all three CPGs were > 70% in domain 4 (AAP = 78%, JSPN = 90%, KDIGO = 100%).

### Domain 5: applicability

The AGREE II standardized scores for domain 5 ranged from 16 to 22%. None of the included CPGs scored > 70%.

### Domain 6: editorial independence

The AGREE II standardized scores for domain 6 ranged from 65 to 94%. The scores of two CPGs were > 70% in domain 6 (AAP = 88%, KDIGO = 94%).

### Overall assessment

The AGREE II standardized domain scores for the first overall assessment ranged from 58 to 75%. Two CPGs scored > 70% (KDIGO and JSPN), which was consistent with the high scores in the six AGREE II domains.

### Recommending the childhood SSNS CPGs for use in practice

The second (overall) assessment (i.e., recommendation for using the CPG in practice) revealed a consensus between the reviewers on recommending the use of two CPGs.

All included CPGs cited SRs in their reference list. The largest number of SR citations was observed in the JPNS CPG (*n* = 12), among them were six Cochrane SRs [26–28], followed by KDIGO–Chapter 3 (*n* = 5) including four Cochrane SRs [29], and lastly AAP (*n* = 4) with one Cochrane SR [25].

## Discussion

Although several regional and national guidelines have been published [25–30], shared treatment guidelines are limited in Saudi Arabia, and consequently, physicians rely on the clinical expertise of each unit to select the best treatment option for pediatric patients with SSNS. To the best of our knowledge, this review is novel in that it systematically evaluates the quality of recently published CPGs of SSNS in children using the AGREE II instrument as a part of a national CPG adaptation initiative.

Three CPGs addressing the management of children with SSNS were assessed using the AGREE II instrument. This AGREE II assessment highlighted several areas of improvement in the methodological rigor of the included CPGs. Although the assessment of overall guideline quality and recommendation for use are standard components of AGREE II, it is possible that they are underreported: standardized domain scores for the first overall assessment ranged from 58 to 75%, with the KDIGO and JSPN scoring > 70%.

In this review, the scores of all three CPGs were > 70% in domain 4. However, it was previously suggested that domain 3 was the strongest indicator of guideline quality than other domains [31–33], with a high score signifying a low degree of bias and evidence-based guideline development [33]. Conversely, a low score suggests serious methodological flaws. This may be the case, for example, if the team in charge of developing the guideline includes experts with little experience in guideline development or if the systematic search strategy is inadequately described [31].

A summary for the key recommendations of the three included CPGs is presented in Tables 3 and 4. A separate classification for the quality of evidence and strength of recommendations has been provided in additional file 4.

**Table 3:** Summary comparison between the three included clinical practice guidelines for management of steroid-sensitive nephrotic syndrome in children): Case definition

Options of care and management of children with SSNS	AAP CPG 2009 [25] Moderate-quality CPG (Domain 3: 40–69%)	JSPN CPG 2014 [26–28] High-quality CPG (Domain 3: ≥ 70%)	KDIGO CPG 2012 [29] High-quality CPG (Domain 3: ≥ 70%)
**Case definition**			
▪ **Nephrotic syndrome**	A urine protein/creatinine ratio (Up/c) of ≥2 and a serum albumin level of ≤2.5 mg/dL	Severe proteinuria (≥40 mg/m^2^/h in pooled night urine) or early morning urine protein/creatinine ratio ≥2.0 g/gCr and hypoalbuminemia (serum albumin level ≤2.5 g/dL)	Presence of the following: ▪ Edema ▪ uPCR ≥2000 mg/g (≥200 mg/mmol) or ≥300 mg/dL or 3+ protein on urine dipstick ▪ Hypoalbuminemia ≤2.5 g/dl (≤25 g/L)
▪ **Remission**	Up/c < 0.2 or Albustix-negative (Albustix, Miles, Inc, Diagnostics Division, Elkhart, IN) or trace for 3 days	• **Complete** Negative protein on dipstick testing of early morning urine for 3 consecutive days or early morning urine protein/creatinine ratio < 0.2 g/gCr for 3 consecutive days • **Incomplete** ≥ 1+ protein on dipstick testing of early morning urine or early morning urine protein creatinine ratio ≥ 0.2 g/gCr and serum albumin > 2.5 g/dL	• **Complete remission**: uPCR < 200 mg/g (< 20 mg/mmol) or < 1+ of protein on urine dipstick for 3 consecutive days • **Partial remission**: Proteinuria reduction ≥ 50% from the presenting value and absolute uPCR between 200 and 2000 mg/g (20–200 mg/mmol)
▪ **Relapse**	After remission, an increase in the first morning Up/c to ≥ 2 or Albustix reading of ≥ 2 for 3 of 5 consecutive days	≥ 3+ protein on dipstick testing of early morning urine for 3 consecutive days	uPCR ≥ 2000 mg/g (≥ 200 mg/mmol) or ≥ 3+ protein on urine dipstick for 3 consecutive days
▪ **FRNS**	Two or more relapses within 6 months after initial therapy or four or more relapses in any 12-month period	Two or more relapses within 6 months after initial remission or four or more relapses within any 12 consecutive months	Two or more relapses within 6 months of initial response or four or more relapses in any 12-month period
▪ **SDNS**	Relapse during taper or within 2 weeks of discontinuation of steroid therapy.	Two consecutive relapses during prednisolone tapering or within 14 days after discontinuation of prednisolone	Two consecutive relapses during corticosteroid therapy or within 14 days of therapy discontinuation
▪ **SRNS**	Inability to induce a remission with 4 weeks of daily steroid therapy	Absence of complete remission after at least 4 weeks of daily prednisolone therapy	No remission after a minimum of 8 weeks treatment with corticosteroids
**Genetic testing**	***Not mentioned***	• Useful in genetic illnesses (***type of testing not mentioned***)	***Not mentioned***

*AAP* American Academy of Pediatrics; *CPGs* clinical practice guidelines; *CNI* calcineurin inhibitor; *CPG ID* short identity or acronym; *JSPN* Japanese Society of Paediatric Nephrology; *CNIs* KDIGO, Kidney Disease: Improving Global Outcomes; *AAP 2009 CPG* Management of childhood onset nephrotic syndrome; *JPNS 2014 CPG* evidence-based clinical practice guidelines for nephrotic syndrome; *KDIGO 2012 CPG* clinical practice guideline for glomerulonephritis—Chapter 3; *ISKDC* International Study of Kidney Disease in Children; *MCNS* minimal change nephrotic syndrome; *MMF* mycophenolate mofetil; *FRNS* Frequently relapsing nephrotic syndrome; *SSNS* steroid-sensitive nephrotic syndrome; *SDNS* steroid-dependent nephrotic syndrome; SRNS: steroid resistant nephrotic syndrome

**Table 4:** Summary comparison between the three included clinical practice guidelines for management of steroid-sensitive nephrotic syndrome in children): Treatment

Options of care and management of children with SSNS	AAP CPG 2009 [25] Moderate-quality CPG (Domain 3: 40–69%)	JSPN CPG 2014 [26–28] High-quality CPG (Domain 3: ≥ 70%)	KDIGO CPG 2012 [29] High-quality CPG (Domain 3: ≥ 70%)
**Diet therapy**	**● Low-fat diet:** limit dietary fat to < 30% of calories, saturated fat to < 10% of calories, and < 300 mg/day dietary cholesterol. • **Low-sodium diet** (**LoE**: Not applicable, **GoR**: Opinion-based)	• Sodium restrictions for remission of edema (Not Graded) • The degree of sodium restrictions should be determined based on the status of edema and amount of food intake. • Base protein consumption on the nutrient requirement for healthy children of the same age Base the caloric energy intake on the age of the patient	***Not mentioned***
**Treatment of initial episode of SSNS with corticosteroids**	● Prednisone 2 mg/kg per day for 6 weeks (maximum: 60 mg); **then** ● Prednisone 1.5 mg/kg on alternate days for 6 weeks (maximum: 40 mg). ● No steroid taper is required at the conclusion of this initial therapy. (**LoE**: Not applicable, **GoR**: Opinion-based)	• **ISKDC regimen**: Prednisolone for 8 weeks (Grade B): 1. 60 mg/m^2^/day or 2.0 mg/kg/day in three divided doses daily for 4 weeks (maximum: 60 mg/day), followed by 2. 40 mg/m^2^ or 1.3 mg/kg once in the morning on alternate days for 4 weeks (maximum: 40 mg on alternate days). • **Long-term, tapering regimen**: prednisolone for 3–7 months	**Oral prednisone or prednisolone as a single daily dose (1B) starting:** • **Daily**: 60 mg/m^2^/day or 2 mg/kg/day to a maximum 60 mg/day (1D) for 4–6 weeks (1C) **then**: **Alternate day**: 40 mg/m^2^ or 1.5 mg/kg to a maximum 40 mg (1D) for 2–5 months with tapering of the dose (1B)
**Treatment of relapsing SSNS with corticosteroids**	● Prednisone 2 mg/kg per day until urine protein test results are negative or trace for 3 consecutive days; **then** ●Prednisone 1.5 mg/kg on alternate days for 4 weeks (**LoE:** Not applicable, **GoR:** Opinion-based)	• **Modified ISKDC regimen** 1. 60 mg/m^2^/day or 2.0 mg/kg/day in three divided doses daily until confirmation of the resolution of proteinuria for at least 3 days but not exceeding 4 weeks (maximum: 60 mg/day), followed by 2. 60 mg/m^2^ or 2.0 mg/kg once in the morning on alternate days for 2 weeks (maximum: 60 mg on alternate days), followed by 3. 30 mg/m^2^ or 1.0 mg/kg once in the morning on alternate days for 2 weeks (maximum: 30 mg on alternate days), followed by 4. 15 mg/m^2^ or 0.5 mg/kg once in the morning on alternate days for 2 weeks (maximum: 15 mg on alternate days). • **Long-term, tapering regimen** Should be selected when appropriate. (Not Graded)	**Initially**: Prednisone as a single daily dose 60 mg/m^2^ or 2 mg/kg (maximum: 60 mg/day) until the child has been in complete remission for at least 3 days (2D) **Then**: Prednisone as a single dose on alternate days (40 mg/m^2^ per dose or 1.5 mg/kg per dose: maximum 40 mg on alternate days) for at least 4 weeks (2C)
**Corticosteroid therapy in frequently relapsing (FR) and steroid-dependent (SD) SSNS in children**	**Frequently relapsing SSNS** ● Prednisone 2 mg/kg/day until proteinuria normalizes for 3 days, 1.5 mg/kg on alternate days for 4 weeks, and then taper over 2 months by 0.5 mg/kg on alternate days (total: 3–4 months). (**LoE:** Not applicable, **GoR:** Opinion-based) **Steroid-dependent SSNS** ● Glucocorticoids are preferred in the absence of significant steroid toxicity. ● Secondary alternatives should be selected based on risk/benefit ratio. (**LoE**: Not applicable, **GoR**: Opinion-based)	Use immunosuppressive agents (e.g., cyclosporine, cyclophosphamide) in the treatment of frequently relapsing and steroid-dependent nephrotic syndrome (Grade C1) due to the development of various steroid-induced side effects.	• **Initially**: daily prednisone until the child has been in remission for at least 3 days • **Then**: alternate-day prednisone for at least 3 months. (2C) **Long term steroid**: prednisone to be given on alternate days in the lowest dose to maintain remission without major adverse effects. (2D) If **not effective**: daily prednisone at the lowest dose to be given to maintain remission without major adverse effects (2D)
Treatment of FR and SD SSNS with corticosteroid-sparing agents			
▪ **Cyclophosphamide**	**Frequently relapsing SSNS** Oral cyclophosphamide 2 mg/kg/day for 12 weeks (cumulative dose: 168 mg/kg) based on ideal body weight started during prednisone (2 mg/kg/day) induced remission, decrease prednisone dose to 1.5 mg/kg on alternate days for 4 weeks, and then taper over 4 weeks. (**LoE**: Not applicable, **GoR**: Opinion-based) **Steroid-dependent SSNS** ● Oral cyclophosphamide 2–3 mg/kg/day for 8–12 weeks. ● Given the severity of cyclophosphamide-associated adverse events, cytotoxic agents are considered a third-line choice for steroid-dependent nephrotic syndrome therapy. (**LoE:** Not applicable, **GoR**: Opinion-based)	• To be given at an initial dose of 2–2.5 mg/kg/day (maximum: 100 mg) and then once daily for 8–12 weeks. (Grade C1) • A second course of cyclophosphamide should not be given and that cumulative doses do not exceed 300 mg/kg.	**Use:** as corticosteroid-sparing agent. (1B) for FR and (2C) for SD SSNS **Dose:** 2 mg/kg/day to be given for 8–12 weeks (maximum cumulative dose 168 mg/kg). (2C) **Timing**: Not to be started until the child has achieved remission with corticosteroids. (2D) **Repeated courses**: second courses of alkylating agents should not be administered. (2D)
▪ **Mycophenolate mofetil (MMF)**	**Frequently relapsing SSNS** Mycophenolate mofetil 25–36 mg/kg/day (maximum: 2 g/day) in two divided doses for 1–2 years with a tapering dose of prednisone. (**LoE**: Not applicable, **GoR**: Opinion-based) **Steroid-dependent SSNS** Mycophenolate mofetil 24–36 mg/kg/day or 1200 mg/m^2^/day in two divided doses (maximum: 2 g/day). (**LoE**: Not applicable, **GoR**: Opinion-based)	• To be considered when standard immunosuppressive agents cannot be used because of their side effects (Grade C1) • A dose of 1000–1200 mg/m^2^/day or 24–36 mg/kg/day (maximum 2 g/day) be administered in two divided doses	**Use**: as corticosteroid-sparing agent (2C) **Dose**: 1200 mg/m^2^/day in two divided doses (2C) **Duration**: at least 12 months (2C)
▪ **Levamisole**	Use of levamisole may reduce the risk of relapses without glucocorticoids. (**LoE**: Not applicable, **GoR**: Opinion-based)	***Not mentioned***	**Use**: as corticosteroid-sparing agent. (1B) **Dose**: 2.5 mg/kg on alternate days (2B) **Duration**: at least 12 months (2C)
▪ **Cyclosporine**	**Frequently relapsing SSNS** ● Cyclosporine A 3–5 mg/kg/day in two divided doses for an average of 2–5 years. ● The nephrotoxic effects of cyclosporine warrant careful monitoring of kidney function and blood drug levels. ● The risk for nephrotoxicity attributable to calcineurin inhibitors makes this a third line option for frequently relapsing nephrotic syndrome. (**LoE:** Not applicable, **GoR**: Opinion-based) **Steroid-dependent SSNS** Cyclosporine A 3–5 mg/kg/day in two divided doses. (**LoE**: Not applicable, **GoR**: Opinion-based)	To be given at an initial dose of 2.5–5 mg/kg/day in two divided doses, followed by dose adjustment according to monitoring of blood drug concentration (Grade C1)	**Use:** as corticosteroid-sparing agent (1C) **Dose**: 4–5 mg/kg/day in two divided doses. (2C) **Monitoring**: Monitor CNI levels during therapy to limit toxicity. (Not Graded) **Duration**: at least 12 months. (2C)
▪ **Mizoribine**	Use of mizoribine (not available in the USA) may reduce the risk of relapses without glucocorticoids. (**LoE**: Not applicable, **GoR**: Opinion-based)	• Not administered at the standard dose (4 mg/kg/day, maximum 150 mg/day) as it would be inadequately effective. (Grade C1) To be administered at higher doses of 7–10 mg/kg/day once daily, with a peak blood mizoribine concentration (C2*^2^ or C3*^3^) ≥ 3.0 μg/mL, because of reported efficacy in preventing relapses	**Not to be used** as corticosteroid sparing agent. (2C)
▪ **Tacrolimus**	**Frequently relapsing SSNS** ● Tacrolimus, an alternative calcineurin inhibitor, provides no advantage regarding nephrotoxicity profile. ● The risk for nephrotoxicity attributable to calcineurin inhibitors makes this a third-line option for frequently relapsing nephrotic syndrome. (**LoE**: Not applicable, **GoR**: Opinion-based) **Steroid-dependent SSNS** Tacrolimus 0.05 to 0.1 mg/kg/day in two divided doses. (**LoE**: Not applicable, **GoR**: Opinion-based)	• To be considered when cyclosporine cannot be used because of its cosmetic side effects. Starting dose (0.1 mg/kg/day) should be administered in two divided doses, followed by dose adjustment according to monitoring of blood drug concentration	**Use**: To be used instead of cyclosporine when the cosmetic side effects of cyclosporine are unacceptable (as corticosteroid-sparing agent). (2D) **Dose**: 0.1 mg/kg/day administered in two divided doses (2D) **Monitoring**: Monitor CNI levels during therapy to limit toxicity. (Not Graded) **Duration**: at least 12 months (2C)
▪ **Chlorambucil**	**Frequently relapsing SSNS** Compared with cyclophosphamide, chlorambucil is associated with a slightly greater toxicity profile and no improvement in efficacy. (**LoE**: Not applicable, **GoR**: Opinion-based) **Steroid-dependent SSNS**: Chlorambucil may reduce the risk of relapses without glucocorticoids. (**LoE**: Not applicable, **GoR**: Opinion-based)	***Not mentioned***	**Use:** as corticosteroid-sparing agent. (1B) for FR and (2C) for SD SSNS **Dose** 0.1–0.2 mg/kg/day may be administered for 8 weeks (maximum cumulative dose 11.2 mg/kg) as an alternative to cyclophosphamide. (2C) **Repeated courses**: second courses of alkylating agents should not be administered (2D)
▪ **Rituximab**	***Not mentioned***	• To be considered only in refractory disease • To be administered at a starting dosage of 375 mg/m^2^ per dose by intravenous drip infusion, administered one to four times (at 1-week intervals for multiple infusions) (Grade C1)	**Use**: to be considered only in children with SD SSNS who have continuing frequent relapses despite optimal combinations of prednisone and corticosteroid- sparing agents and/or who have serious adverse effects of therapy. (2C)
**Indication for kidney biopsy**	● A kidney biopsy for children aged ≥ 12 years is recommended because of the frequency of diagnoses other than minimal-change disease. (**LoE**: Not applicable, **GoR:** Opinion-based)	• At the onset of nephrotic syndrome in patients: (Not Graded): 1. Whose age is < 1 year 2. With persistent hematuria and frank hematuria 3. Hypertension and renal dysfunction 4. Hypocomplementemia 5. Extrarenal symptoms (e.g., rash, purpura), since these patients are likely to have other histological types than minimal-change disease. • In patients showing steroid resistance • In patients given long-term calcineurin inhibitor therapy, even without renal dysfunction (at 2–3 years into the therapy)	Indications for kidney biopsy in children with SSNS are (Not Graded): ▪ Late failure to respond following initial response to corticosteroids ▪ A high index of suspicion for a different underlying pathology ▪ Decreasing kidney function in children receiving CNIs
**Vaccination in children with SSNS**	● Immunize with the 23-valent and heptavalent conjugated pneumococcal vaccines. ● Immunize the immunosuppressed or actively nephrotic patient and household contacts with inactivated influenza vaccine yearly. ● Defer immunization with live vaccines: - Until prednisone dose is < 2 mg/kg/day (maximum: 20 mg). - For 3 months from completion of therapy with cytotoxic agents or for 1 month from completion of other daily immunosuppression. ● Provide varicella immunization if nonimmune based on immunization history, disease history, or serologic evaluation. ● Provide postexposure immunoglobulin for nonimmune immunocompromised patients. ● Consider intravenous acyclovir for immunosuppressed children at the onset of chicken pox lesions. (**LoE**: Not applicable, **GoR**: Opinion-based)	• Perform immunizations, when applicable. • Not use live attenuated vaccines in patients during steroid or immunosuppressant treatment. • Attenuated vaccines may be determined on a case-by-case basis and according to the condition of the patient and epidemic (Grade B) • Proactive vaccination to the family member of the patient if there is no history or vaccination against the prevalent infection prophylaxis with antiviral drugs (acyclovir or valaciclovir) in cases where the household has been in close contact with varicella	**To reduce the risk of serious infections in children with SSNS (Not Graded):** ▪ Provide pneumococcal vaccination to the children. ▪ Provide influenza vaccination annually to the children and their household contacts. ▪ Defer vaccination with live vaccines until prednisone dose is below either 1 mg/kg daily (< 20 mg/day) or 2 mg/kg on alternate days (< 40 mg on alternate days). ▪ Live vaccines are contraindicated in children receiving corticosteroid-sparing immunosuppressive agents. ▪ Immunize healthy household contacts with live vaccines to minimize the risk of transfer of infection to the immunosuppressed child but avoid direct exposure of the child to gastrointestinal, urinary, or respiratory secretions of vaccinated contacts for 3–6 weeks after vaccination. ▪ Following close contact with varicella infection, administer varicella zoster immune globulin, if available, to nonimmune children on immunosuppressive agents
**Relevant implementation tool(s) provided in the CPG**	Table 1. Monitoring recommendations for children with nephrotic syndrome	• Fig. 1. Flowchart for the determination of treatment plan [27] • Table 5. Examination findings of primary nephrotic syndrome [26] • Fig. 1. Treatment of MCNS [26] • Table 1. Diuretic agents available for infants/children [28] • Table 2. 2Dietary reference intake for Japanese population [28] • Table 3. Health classification by the status of nephrotic syndrome [28]	Translations into four languages: Japanese, German, Russian, and Turkish. The Canadian Society of Nephrology published a Commentary in 2014 on the KDIGO 2012 CPG (management of nephrotic syndrome in children) including the relevancy and applicability of the recommendations to the Canadian context.

*AAP* American Academy of Pediatrics; *CPGs* clinical practice guidelines; *CNI* calcineurin inhibitor; *CPG ID* short identity or acronym; *GoR* grade of recommendation; *JSPN* Japanese Society of Paediatric Nephrology; *KDIGO* Kidney Disease: Improving Global Outcomes; *LoE* Level (or quality) of evidence; *FRNS* Frequently relapsing nephrotic syndrome, *SSNS* steroid-sensitive nephrotic syndrome; *AAP 2009 CPG* management of childhood onset nephrotic syndrome; *JPNS 2014 CPG* evidence-based clinical practice guidelines for nephrotic syndrome; *KDIGO 2012 CPG* clinical practice guideline for glomerulonephritis—Chapter 3; *ISKDC* International Study of Kidney Disease in Children; *MCNS* minimal change nephrotic syndrome; *MMF* mycophenolate mofetil

These key elements of the management of childhood SNSS included case definition, genetic testing, diet therapy, treatment of an initial episode of SSNS with steroids, treatment of relapsing SSNS with steroids, steroid therapy in FRNS and SDNS in children, treatment of FRNS and SDNS with steroid-sparing agents, renal biopsy, and vaccination in these children.

A set of strengths were noted in our work. First, we used a comprehensive search strategy to identify potentially relevant CPGs and performed quality assessment using the AGREE II tool by an expert specialized clinical team of pediatric nephrologists, general pediatricians, a clinical pharmacist, a renal pathologist, and a specialized nurse guided by two pediatricians with expertise in CPG methodologies, which adds a layer of strength to the AGREE II assessment. The results of this review can be used as a basis for CPG development or adaptation projects for the management of children with SSNS.

Furthermore, the results of our study propose the importance of including the AGREE II criteria in the capacity building of clinicians to guide their decisions in selecting CPGs for use in their daily practice.

Our study also has several limitations. First, some disadvantages of AGREE II have been addressed in the “AGREE-REX” (Recommendation EXcellence) tool, which addresses the clinical credibility of the CPG recommendations [31]. The selection of 70% as a cutoff point for standard domain ratings is another potential limitation as the original AGREE II does not mandate such a cutoff, but similar studies have also suggested so [22, 23].

Other limitations, apart from those imposed by the AGREE II, include the following: (i) only English or Arabic language CPGs may have resulted in the exclusion of relevant CPGs intended for use in non-English-speaking and non-Arabic healthcare settings; (ii) this review mainly focused on CPGs for management of SSNS in children and did not evaluate other subcategories of nephrotic syndrome in children as it was out of the scope of this study.

### Implications for practice: guidance for clinical guideline uptake

The adaptation of CPGs has been identified as a valid alternative to de novo development, which is a resource-extensive process [13]. Evidence-based practice initiatives in several countries in our region have opted to utilize CPG adaptation rather than de novo development [11, 12]. Several CPG formal adaptation methodologies are presently available and could be further customized to local contexts [13]. Studies similar to our study could provide information on relevant CPG adaptation projects for the same health topics, especially for groups with little experience in using the AGREE II instrument.

This critical appraisal highlights the importance of quality assessment of CPGs by clinicians to ensure the transparency and strength of the CPG development process according to international CPG standards and support the best practice for children with SNSS. We recommend incorporating the AGREE II appraisal of CPGs in the capacity building of pediatricians and nephrologists.

## Conclusions

The methodological quality of the KDIGO CPG was superior, followed by JSPN and AAP CPGs. Recommendations including the case definition, genetic testing, diet therapy, treatment of an initial episode of SSNS with steroids, treatment of relapsing SSNS with steroids, steroid therapy in FRNS and SDNS in children, treatment of FRNS and SDNS with steroid-sparing agents, renal biopsy, and vaccination in children with SSNS.

## Supplementary Information

**Additional file 1.** Search strategy.

**Additional file 2.** PICAR statement.

**Additional file 3.** PRISMA checklist.

**Additional file 4.** Evidence-Base Classifications.

## Data Availability

Not applicable.

## Abbreviations

AGREE: Appraisal of Guidelines for REsearch & Evaluation
AAP: American Academy of Pediatrics
CPG: Clinical Practice Guideline
JSPN: Japanese Society of Pediatric Nephrology
KDIGO: Kidney Disease: Improving Global Outcomes
SR: Systematic review
SSNS: Steroid-sensitive nephrotic syndrome
