# Supplementary material for: AGREEing on clinical practice guidelines for idiopathic steroid-sensitive nephrotic syndrome in children
Source: Syst Rev. 2021 May 10;10:144. doi: 10.1186/s13643-021-01666-w (PMC8112064; doi:10.1186/s13643-021-01666-w)
Supplement: Supplementary file 1 — Additional file 1. Search strategy. [file 13643_2021_1666_MOESM1_ESM.docx]

**S1 Supporting information.**

**Search strategy for bibliographic databases**

**Embase**1996 to 2019 November 01

| 1 |  | exp nephrotic syndrome/ | 15749 |  |
| --- | --- | --- | --- | --- |
| 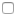 | 2 | steroid dependent nephrotic syndrome.mp. | 412 |  |
| 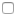 | 3 | steroid resistant nephrotic syndrome.mp. | 1240 |  |
| 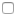 | 4 | child*.mp. | 1940025 |  |
| 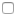 | 5 | p??diatric*.mp. | 533280 |  |
| 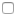 | 6 | exp pediatrics/ | 83294 |  |
| 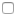 | 7 | exp practice guideline/ | 513176 |  |
| 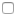 | 8 | exp Organizational Policy/ or exp Guideline Adherence/ or exp.mp. [mp=title, abstract, heading word, drug trade name, original title, device manufacturer, drug manufacturer, device trade name, keyword, floating subheading word, candidate term word] | 24715 |  |
| 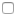 | 9 | Reference Standards/ or exp Guidelines as Topic/ or exp Practice Guidelines as Topic/ or guidelin*.mp. | 988089 |  |
| 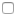 | 10 | 1 or 2 or 3 | 16257 |  |
| 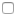 | 11 | 4 or 5 or 6 | 2069968 |  |
| 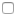 | 12 | 7 or 8 or 9 | 1006055 |  |
| 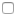 | 13 | 10 and 11 and 12 | 286 |  |
| 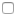 | 14 | 13 | 286 |  |
| 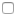 | 15 | limit 13 to (english language and yr="2009 -Current") | 195 |  |

**Ovid MEDLINE(R)**1996 to November 01, 2019**,****Ovid MEDLINE(R) and Epub Ahead of Print, In-Process & Other Non-Indexed Citations and Daily - without Revisions**2015 to November 01, 2019

| 1 | 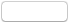 | exp nephrotic syndrome/ | 10195 |  |
| --- | --- | --- | --- | --- |
| 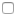 | 2 | 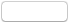  steroid dependent nephrotic syndrome.mp. | 339 |  |
| 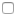 | 3 | 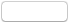  steroid resistant nephrotic syndrome.mp. | 1012 |  |
| 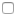 | 4 | 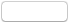  child*.mp. | 1980825 |  |
| 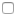 | 5 | 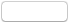  p??diatric*.mp. | 406988 |  |
| 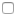 | 6 | 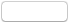  exp pediatrics/ | 48006 |  |
| 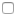 | 7 | 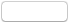  exp practice guideline/ | 29079 |  |
| 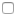 | 8 | 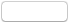  exp Organizational Policy/ or exp Guideline Adherence/ or exp.mp. [mp=title, abstract, original title, name of substance word, subject heading word, floating sub-heading word, keyword heading word, organism supplementary concept word, protocol supplementary concept word, rare disease supplementary concept word, unique identifier, synonyms] | 60906 |  |
| 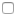 | 9 | 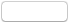  Reference Standards/ or exp Guidelines as Topic/ or exp Practice Guidelines as Topic/ or guidelin*.mp. | 552149 |  |
| 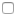 | 10 | 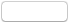  1 or 2 or 3 | 10553 |  |
| 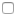 | 11 | 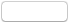  4 or 5 or 6 | 2070633 |  |
| 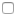 | 12 | 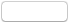  7 or 8 or 9 | 573144 |  |
| 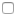 | 13 | 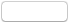  10 and 11 and 12 | 89 |  |
| 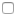 | 14 | 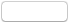  13 | 89 |  |
| 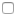 | 15 | 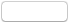  limit 13 to (english language and yr="2009 -Current") | 57 |  |
