# Supplementary material for: AGREEing on clinical practice guidelines for idiopathic steroid-sensitive nephrotic syndrome in children
Source: Syst Rev. 2021 May 10;10:144. doi: 10.1186/s13643-021-01666-w (PMC8112064; doi:10.1186/s13643-021-01666-w)
Supplement: Supplementary file 2 — Additional file 2. PICAR statement. [file 13643_2021_1666_MOESM2_ESM.docx]

**Elements of the PICAR statement of relevance to SSNS CPGs**

| **PICAR Item** | **PICAR items relevant to screening CPGs for**  **inclusion** |
| --- | --- |
| **P: Population, clinical indication(s), and**  **condition(s)** | **Study population**: Children aged 2–12 years.  **Clinical indication:** Treatment (pharmacological therapeutic agents).  **Clinical condition:** Non-congenital, idiopathic SSNS, including new-onset nephrotic syndrome, SDNS, or FRNS without any comorbidities |
| **I: Intervention(s)** | All interventions are of interest mainly pharmacological therapeutic agents**.** |
| **C: Comparator(s), comparison(s), and (key)**  **content** | **The following Key clinical content is of interest in eligible CPGs: -**   1. Treatment of initial episode of SSNS with CSs. 2. Treatment of relapsing SSNS with corticosteroids. 3. CS therapy in frequently relapsing (FR) and steroid-dependent (SD) SSNS in children. 4. Indication for kidney biopsy. 5. Vaccination in children with SSNS |
| **A: Attributes of the CPG** | **CPG eligibility (inclusion) criteria:** -   1. Evidence-based with a clear record of their development methods 2. Published in English or Arabic language. 3. Original source CPGs (de novo development). 4. National or international scope and purpose. 5. Published by an organization or group authorship and accessible from a CPG database or peer-reviewed journal. Only the most current version of each source CPG was appraised.   **CPG exclusion criteria: -**   1. Published earlier than 2009. 2. Not in the English language. 3. Adapted from other CPGs. 4. Proposed as consensus or expert-based statements. 5. single author CPG. |
| **R: Recommendation characteristics and**  **‘‘other’’ considerations** | CPG eligibility is dependent on the presence of eligible CPG Recommendations for CS therapy and regimens for SSNS initial episodes and relapses. In addition to CS-sparing agents for FRNS and SDNS. |

**Abbreviations:** CPGs, clinical practice guidelines; CS: Corticosteroid, FRNS: Frequently relapsing nephrotic syndrome; SSNS: steroid-sensitive nephrotic syndrome; SDNS: steroid-dependent nephrotic syndrome; SRNS: steroid resistant nephrotic syndrome
