# Supplementary material for: AGREEing on clinical practice guidelines for idiopathic steroid-sensitive nephrotic syndrome in children
Source: Syst Rev. 2021 May 10;10:144. doi: 10.1186/s13643-021-01666-w (PMC8112064; doi:10.1186/s13643-021-01666-w)
Supplement: Supplementary file 4 — Additional file 4. Evidence-Base Classifications. [file 13643_2021_1666_MOESM4_ESM.docx]

**Additional file 4 Classification of the level/ quality of evidence and grade/ strength of recommendations for the three eligible clinical practice guidelines**

| **CPG ID** | **Level/ Quality of Evidence** | **Definition** | **Grade/ Strength of Recommendation** | **Definition** |
| --- | --- | --- | --- | --- |
| **KDIGO 2012 CPG** [29] | | | | |
|  | **A (High)** | We are confident that the true effect lies close to that of the estimate of the effect | **Level 1 ‘‘We recommend’’** | **Implications: For patients:** Most people in your situation would want the recommended course of action and only a small proportion would not. **For clinicians:** Most patients should receive the recommended course of action. **For Policy:** The recommendation can be evaluated as a candidate for developing a policy or a performance measure. |
|  | **B (Moderate)** | The true effect is likely to be close to the estimate of the effect, but there is a possibility that it is substantially different. | **Level 2 ‘‘We suggest’’** | **Implications: For patients:** The majority of people in your situation would want the recommended course of action, but many would not. **For clinicians:** Different choices will be appropriate for different patients. Each patient needs help to arrive at a management decision consistent with her or his values and preferences. **For Policy:** The recommendation is likely to require substantial debate and involvement of stakeholders before policy can be determined. |
|  | **C (Low)** | The true effect may be substantially different from the estimate of the effect. | **Not Graded** | The additional category ‘‘Not Graded’’ was used, typically, to provide guidance based on common sense or where the topic does not allow adequate application of evidence. The most common examples include recommendations regarding monitoring intervals, counselling, and referral to other clinical specialists. The ungraded recommendations are generally written as simple declarative statements, but are not meant to be interpreted as being stronger recommendations than Level 1 or 2 recommendations. |
|  | **D (Very low)** | The estimate of effect is very uncertain, and often will be far from the truth. |  |  |
| **JSPN 2014 CPG** [26-28] | | | | |
|  | **Level 1** | Evidence from review articles or meta-analysis articles | **Grade A** | There is strong scientific evidence that intervention is beneficial, and intervention is strongly recommended |
|  | **Level 2** | Evidence from randomized controlled trials | **Grade B** | There is scientific evidence that intervention is beneficial, and intervention is recommended |
|  | **Level 3** | Evidence from non-randomized controlled trials, non-  controlled trials (i.e., single-arm prospective  interventional trials) | **Grade C1** | There is no scientific evidence that intervention is beneficial, but intervention is suggested |
|  | **Level 4** | Evidence from cohort studies, case–control studies, cross-  sectional studies, comparative observational studies, non-  comparative observational studies | **Grade C2** | There is no scientific evidence that intervention is beneficial, and it is recommended not to conduct any intervention |
|  | **Level 5** | Evidence from accumulated cases, case reports, or others  (e.g., descriptive studies) | **Grade D** | There is scientific evidence that intervention is ineffectiveness or harmful, and it is recommended  not to conduct any intervention |
|  | **Level 6** | Evidence from expert committee reports or personal  opinions of experts that are not based on patient data |  |  |
| **AAP 2009 CPG** [25] | | | | |
|  | Not Applicable. | Clear evidence-based CPGs | Opinion-based or consensus-based | Recommendations were largely consensus-based or expert opinion by the physician participants when the literature was insufficient at the time of publication. |
